# Targeted therapy for intractable cancer on the basis of molecular profiles: An open-label, phase II basket trial (Long March Pathway)

**DOI:** 10.3389/fonc.2023.860711

**Published:** 2023-02-23

**Authors:** Xiao-Dong Jiao, Bao-Dong Qin, Zhan Wang, Ke Liu, Ying Wu, Yan Ling, Wen-Xing Qin, Miao-Miao Wang, Ling-Yan Yuan, Savio George Barreto, Anthony W. Kim, Kimberley Mak, Hao Li, Yuan-Yuan Xu, Xiao-Ming Qiu, Min Wu, Min Jin, Li-Chao Xu, Yi Zhong, Hui Yang, Xue-Qin Chen, Yu Zeng, Jun Shi, Wen-Yu Zhu, Qing-Qing Ding, Wei Jia, Su-Fen Liu, Jun-Jing Zhou, Hong Shen, Shi-Hua Yao, Zhao-Ji Guo, Ting Li, Pei-Juan Zhou, Xue-Wei Dong, Wen-Feng Lu, Robert L. Coleman, Mehmet Akce, Chérif Akladios, Francesco Puccetti, Yuan-Sheng Zang

**Affiliations:** ^1^ Department of Medical Oncology, Changzheng Hospital, Naval Medical University, Shanghai, China; ^2^ Department of Surgery, Flinders Medical Centre, Bedford Park, SA, Australia; ^3^ Division of Thoracic Surgery, Keck School of Medicine of the University of Southern California, Los Angeles, CA, United States; ^4^ Department of Radiation Oncology, Boston Medical Center, Boston University School of Medicine, Boston, MA, United States; ^5^ Department of Medical Oncology, Shanghai Ruijin Hospital, Shanghai Jiaotong University, Shanghai, China; ^6^ Department of Surgical Oncology, Shanghai Chest Hospital, Shanghai Jiao Tong University, Shanghai, China; ^7^ Lung Cancer Center, West China Hospital, Sichuan University, Chengdu, China; ^8^ Department Gynecology and Obstetrics, Union Hospital, Tongji Medical College, Huazhong University of Science and Technology, Wuhan, China; ^9^ Cancer Center, Union Hospital, Tongji Medical College, Huazhong University of Science and Technology, Wuhan, China; ^10^ Department of Interventional Radiology, Shanghai Cancer Center, Fudan University, Shanghai, China; ^11^ Department of Medical Oncology, Shanghai Traditional Chinese Medicine-Integrated Hospital, Shanghai University of Traditional Chinese Medicine, Shanghai, China; ^12^ Department of Medical Oncology, Suzhou Municipal Hospital, Nanjing Medical University, Suzhou, China; ^13^ Department of Medical Oncology, Hangzhou First People’s Hospital, Zhejiang University School of Medicine, Hangzhou, China; ^14^ Department of Pathology, Shanghai Tongji Hospital, Shanghai Tongji University, Shanghai, China; ^15^ Department of Gastrointestinal Surgery, Changzhou No.2 People’s Hospital, Nanjing Medical University, Changzhou, China; ^16^ Department of Medical Oncology, Changzhou No.2 People’s Hospital, Nanjing Medical University, Changzhou, China; ^17^ Department of Geriatric Oncology, Jiangsu Provincial People’s Hospital, Nanjing Medical University, Nanjing, China; ^18^ Department of Respiratory, Shanghai Traditional Chinese Medicine-Integrated Hospital, Shanghai University of Traditional Chinese Medicine, Shanghai, China; ^19^ Department of Gynecology, Changzhou No.2 People’s Hospital, Nanjing Medical University, Changzhou, China; ^20^ Department of Hepatobiliary and Pancreatic Surgery, Wuxi No.4 People’s Hospital, Jiangnan University, Wuxi, China; ^21^ Department of Medical Oncology, Second Affiliated Hospital of Zhejiang University School of Medicine, Hangzhou, China; ^22^ Department of Thoracic Surgery, Changhai Hospital, Naval Medical University, Shanghai, China; ^23^ Department of General Surgery, The First Affiliated Hospital of Soochow University, Suzhou, China; ^24^ Department of Medical Oncology, Shanghai Cancer Center, Fudan University Shanghai Cancer Center, Shanghai, China; ^25^ Department of Traditional Chinese Medicine, Shanghai Renji Hospital, Shanghai Jiaotong University, Shanghai, China; ^26^ Department of Gastrointestinal Surgery, The First People’s Hospital of Changzhou, Soochow University, Changzhou, China; ^27^ Department of Integrative Medicine, Zhongshan Hospital, Fudan University, Shanghai, China; ^28^ Department of Gynecologic Oncology and Reproductive Medicine, The University of Texas MD Anderson Cancer Center, Houston, TX, United States; ^29^ Department of Hematology and Medical Oncology, Winship Cancer Institute of Emory University, Atlanta, GA, United States; ^30^ Department of Obstetrics and Gynecology, University of Strasbourg, Strasbourg, France; ^31^ Department of Gastrointestinal Surgery, San Raffaele Hospital IRCCS, Milan, Italy

**Keywords:** intractable cancer, basket trial, targeted therapy, gene alteration, precision medicine

## Abstract

**Purpose:**

We evaluated he effects of molecular guided-targeted therapy for intractable cancer. Also, the epidemiology of druggable gene alterations in Chinese population was investigated.

**Materials and methods:**

The Long March Pathway (ClinicalTrials.gov identifier: NCT03239015) is a non-randomized, open-label, phase II trial consisting of several basket studies examining the molecular profiles of intractable cancers in the Chinese population. The trial aimed to 1) evaluate the efficacy of targeted therapy for intractable cancer and 2) identify the molecular epidemiology of the tier II gene alterations among Chinese pan-cancer patients.

**Results:**

In the first stage, molecular profiles of 520 intractable pan-cancer patients were identified, and 115 patients were identified to have tier II gene alterations. Then, 27 of these 115 patients received targeted therapy based on molecular profiles. The overall response rate (ORR) was 29.6% (8/27), and the disease control rate (DCR) was 44.4% (12/27). The median duration of response (DOR) was 4.80 months (95% CI, 3.33−27.2), and median progression-free survival (PFS) was 4.67 months (95% CI, 2.33−9.50). In the second stage, molecular epidemiology of 17,841 Chinese pan-cancer patients demonstrated that the frequency of tier II gene alterations across cancer types is 17.7%. Bladder cancer had the most tier-II alterations (26.1%), followed by breast cancer (22.4%), and non-small cell lung cancer (NSCLC; 20.2%).

**Conclusion:**

The Long March Pathway trial demonstrated a significant clinical benefit for intractable cancer from molecular-guided targeted therapy in the Chinese population. The frequency of tier II gene alterations across cancer types supports the feasibility of molecular-guided targeted therapy under basket trials.

## Introduction

Oncogenic alterations could result in the promotion of carcinogenesis and cancer progression (1). Development of therapy targeting druggable mutations (potentially actionable with an already approved therapy) enables patients with these gene alterations to experience significantly improved clinical benefits compared to conventional therapy (e.g., targeting *HER-2* overexpression in the breast or gastric cancer, *BRAF V600E* mutation in melanoma, *EGFR* or *ALK* in lung cancer) (2–4). The choice of therapy guided by an individual’s tumor molecular profile has become the standard of care for these cancer types. It has been proposed that druggable gene alterations may occur across a wide variety of tumor types due to the advancements in sequencing technologies and a deeper understanding of gene alterations in different tumor types (5). The unanswered question is whether regimens that target specific gene alterations can induce responses regardless of the cancer histology, or location.

To this end, a novel clinical trial design referred to as a “basket trial”, was developed. This study design focuses on specific gene alterations found in tumors, regardless of the location of cancer origins (6–8). Based on prior basket trials investigating the presence of specific gene alterations, a few of therapeutic strategies have been approved for pan-cancer patients independent of cancer types (i.e., larotrectinib for neurotrophic tyrosine kinase, receptor, type (NTRK) 1–3 fusion, pembrolizumab for mismatch repair high deficiency/microsatellite instability, and pembrolizumab for high tumor mutation burden [*TMB*]; >10 mut/Mb]) (9–11).

Intractable cancers are challenging to treat since they do not respond to currently approved therapies. Also included under the broad umbrella of intractable cancers are advanced cancers that have been treated (unsuccessfully) with multiple lines of therapy, as well as those without an effective standard of care (including rare cancers) (12). There is early evidence to suggest that basket trials may provide more clinical benefits than best supportive care for intractable cancers. Hyman et al. (13, 14) conducted a basket trial of 120 refractory cancer patients harboring *BRAF* mutations, all of whom received vemurafenib. In non-small cell lung cancer (NSCLC) patients, the objective response rate (ORR) was 42% after multiline therapy, which is a more favorable response when compared with an ORR of 7% reported for standard, second-line treatment with docetaxel. In a cholangiocarcinoma cohort, the disease control rate (DCR) was 62%, which was also better than second-line chemotherapy (49.5%) (15). In addition to single-gene alterations, basket trials targeting multiple gene alterations have also been conducted for intractable cancers (the MyPathway study) (16).

Previous basket trials have mainly focused on non–East Asian populations. It is known that gene alterations driving cancer progression differ significantly across ethnicities (17). Both the effects of targeted therapy on the basis of molecular profiles for intractable cancer and the molecular epidemiology of druggable gene alterations in the Chinese population remain unclear. The present study aimed to evaluate the efficacy of molecular-guided targeted therapy for intractable cancer among 520 patients from a single cancer center, then further to identify the molecular epidemiology of druggable gene alterations among another 17,841 pan-cancer patients from 24 cancer centers in China.

The druggable gene alterations were defined to be either tier I gene alterations or tier II gene alterations. Tier I gene alterations have the strongest clinical significance, and are supported by level A evidence (Food and Drug Administration [FDA]–approved therapy included in professional guidelines) and level B evidence (well-powered studies with consensus from experts in the field). Tier II gene alterations are of potential clinical significance, and are supported by level C evidence (FDA-approved therapies for different tumor types or investigational therapy, multiple small published studies with some consensus) and level D evidence (preclinical trials or a few cases report without consensus) (18).

## Materials and methods

### Trial design and procedure

The Long March Pathway (ClinicalTrial.gov identifier: NCT03239015) is a non-randomized, open-label, phase II trial that combines multiple basket studies of intractable pan-cancer patients from China. The trial was conducted in 2 main stages. The first part of the Long March Pathway trial included 2 steps. In the first step, 520 intractable cancer patients were recruited prospectively to identify druggable gene alterations. Intractable cancer patients, considered to be those patients who had no standard of care according to the National Comprehensive Cancer Network (NCCN) guidelines (i.e., those with advanced solid tumor after multiple-line therapy, those unresponsive to the standard of care, those ineligible for standard care because of poor performance status) older than 18 years with any type of metastatic solid tumor were eligible for this trial. Additional eligibility requirements included measurable, or evaluable, lesions (19).

Genetic profiling was determined using a next-generation sequencing (NGS) platform (Burning Rock Biotech, Guangzhou, China) in a clinical laboratory improvement amendment (CLIA)–approved laboratory. The protocols were described previously (20, 21). Briefly, genetic profiling was performed using tumor tissue from the most recent tumor biopsy or circulating tumor DNA (ctDNA). DNA was extracted and the NGS library was prepared. Target capture was performed using a panel consisting of 520 cancer-related genes (OncoScreen Plus 520) spanning 1.64 megabases of the human genome. Indexed samples were sequenced on Nextseq 500 (Illumina, Inc., CA, USA) with paired-end reads and an average sequencing depth of 1,000× for tissue samples and 10,000× for liquid biopsy samples. Sequencing data were mapped to the human genome (hg19) using Burrows-Wheeler aligner 0.7.10 (22). Tissue and plasma samples were compared against their own white blood cell control to identify somatic variants. Variants were filtered using the VarScan fpfilter pipeline. Base calling in plasma and tissue samples required at least 8 supporting reads for single nucleotide variations (SNVs) and 2 and 5 supporting reads for insertion-deletion variations (Indels), respectively. Single nucleotide polymorphisms (SNPs) were excluded if population frequency of was over 0.1% in the ExAC, 1000 Genomes, dbSNP or ESP6500SI-V2 databases. Copy number variations (CNVs) were analyzed based on the depth of coverage data of capture intervals.

Patients enrolled in the trial were eligible for the second step of the first stage if 1, or more, druggable gene alterations were identified after genetic profiling screening (e.g., *EGFR* mutation/amplification, *ALK* fusion, *BRAF V600E* mutation, *BRCA1/2* mutation, *CDK4/6* amplification, *CDKN2A/2B* deletion mutation, *ERBB2* amplification/mutations, *FGFR1/2/3* fusion, *MET* amplification, *PIK3CA* mutation, *RET* fusion, *ROS1* fusion).

Based on their options, patients with the above druggable gene alterations were able to receive a molecular-guided targeted therapy. Patients with *ALK* fusions, *MET* amplification, or *ROS1* fusions were treated with crizotinib (250 mg orally twice daily). Patients with *ERBB2* alterations were treated with pyrotinib (400 mg orally once daily). Patients with *CDK4/6* amplifications or *CDKN2A/2B* deletion mutations were treated with palbociclib (125 mg orally once daily for 14 days every 3 weeks). Patients with *BRCA1/2* mutations were treated with olaparib (400 mg orally twice daily). Patients with *BRAF* mutations were treated with vemurafenib (960 mg orally once daily). Patients with *EGFR* mutations were treated with a first-generation EGFR tyrosine kinase inhibitor (TKI; gefitinib 250 mg orally once daily). Patients with *PIK3CA* mutations were treated with everolimus (10 mg orally once daily). Patients with *EGFR* amplifications were treated with nimotuzumab (100 mg intravenous injection once per week). Patients with *FGFR1/2/3* fusions or mutations were treated with erdafitinib (8 mg orally once daily). Patients received treatment for 2 cycles and were then evaluated for treatment response. Patients with objective response or stable disease (SD) continued the targeted therapy, with repeat evaluations every 2 cycles for the first 24 weeks, followed by evaluations every 12 weeks until tumor progression or unacceptable toxicity.

In the second stage of the trial, we conducted a multi-center study to determine the possibility of the above described molecular-guided targeted therapy in pan-cancer. The molecular epidemiology of the druggable gene alterations were evaluated in a larger cohort of 17,841 pan-cancer patients across 16 principal tumors types. In this stage, cancer types included NSCLC, SCLC, breast cancer, colorectal cancer, stomach cancer, liver cancer, pancreatic cancer, biliary system cancer, ovarian cancer, uterus cancer, prostate cancer, bladder cancer, kidney cancer, malignant melanoma, head and neck tumors, and sarcoma. All samples were obtained from fresh tissue or formalin-fixed paraffin-embedded tumor tissue, and they were profiled in a CLIA-certified NGS laboratory using the OncoScreen 295 or OncoScreen Plus 520 cancer-related gene panels (Burning Rock Biotech, Guangzhou, China). Molecular epidemiology of the tier II gene alterations in the pan-cancer patients were also determined based on genetic profiling. The trial was approved by the Ethics Committee of Changzheng Hospital (2017SL016). All participants provided written informed consent to take part in the study.

### Study endpoints

The primary study endpoints for the Long March Pathway trial were ORR. Treatment response was evaluated by the investigator according to Response Evaluation Criteria in Solid Tumors (RECIST) version 1.1 (19). Secondary study endpoints included disease-control rate (DCR), duration of response (DOR), and progression-free survival (PFS). DOR was defined as the time interval from the first documentation of objective response to tumor progression/death, or the most recent tumor assessment. PFS was defined as the time from targeted therapy initiation to death from any cause or progression according to RECIST.

Among the cancer patients who underwent multiple-line therapy, PFS2/PFS1 ratios were also analyzed. PFS2 was defined as the PFS after genomics-guided targeted therapy. PFS on prior therapy (PFS1) was defined as the time interval from the most recent prior treatment to progression (as defined by RECIST), or clinical progression. A PFS2/PFS1 ratio of greater than 1.3 was considered indicative of a treatment benefit, in keeping with the definition of clinical benefit in basket trials (23). Additional secondary study endpoints for the trial were the frequencies of druggable gene alterations outside of tumor-specific approved targeted therapy (tier II gene alterations) among the Chinese pan-cancer patients.

### Statistical analysis

We used a Simon’s minimax 2-stage design with a type I error of 0.10 and 90% power to test the null hypothesis of an ORR of 10% or lower versus the alternative hypothesis of one 30% or higher (24). The regimen would be considered worthy of further investigation if 2 or more of 18 patients achieved partial response (PR) or complete response (CR), after which another 9 patients would be included. Analyses of ORR and DCR were performed based on the efficacy analysis of the population, which included noting those patients with measurable disease who 1) were treated with targeted therapy on the basis of molecular profiles and assessed for clinical response, or 2) had discontinued treatment for any reason (e.g., cancer-related death) before the first response evaluation. DOR analysis was conducted among patients who showed objective responses. The data cutoff date for interim efficacy analysis was September 1, 2020. The Kaplan-Meier curve was drawn to estimate median DOR/PFS with a 95% confidence interval (CI). The frequency of druggable gene alterations across a variety of cancers was defined as the portion of patients with druggable gene alterations who could receive off-label use of a molecular-guided targeted therapy. All statistical analyses were conducted in MedCalc software (MedCalc Software, Mariakerke,Belgium). A *P* value <0.05 was considered statistically significant.

## Results

### Study flow

**Figure 1:**
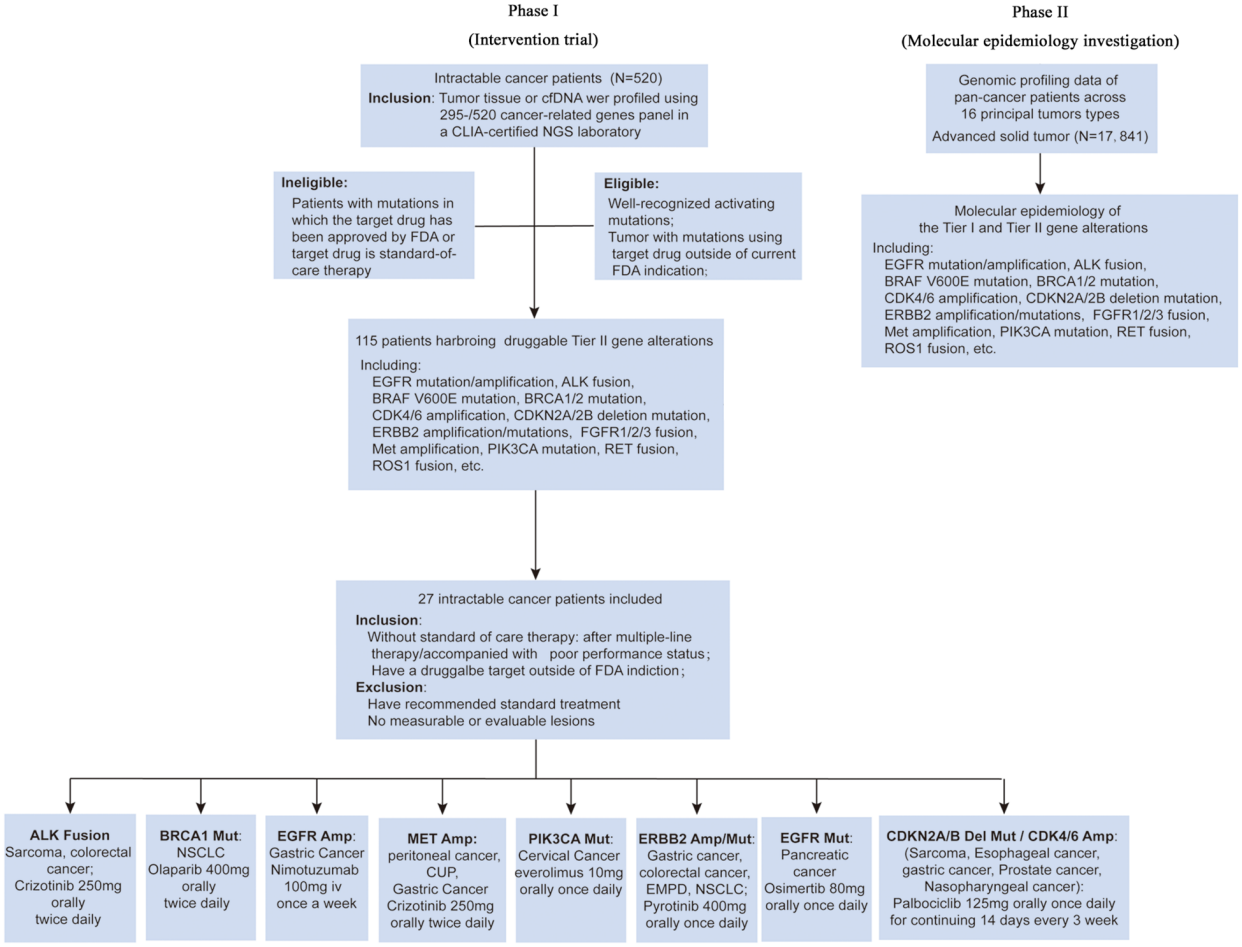
Long March Pathway design.

The study flow is presented in **Figure 1**. In the first phase (Intervention trial), 520 patients were involved in the molecular profiles screening phase between April 2016 and August 2021. Druggable Tier II gene alterations (e.g., *EGFR* mutation/amplification, *ALK* fusion, *BRAF V600E* mutation, *BRCA1/2* mutation, *CDK4/6* amplification, *CDKN2A/2B* deletion mutation, *ERBB2* amplification/mutations, *FGFR1/2/3* fusion, *MET* amplification/mutation, *PIK3CA* mutation, *RET* fusion, and *ROS1* fusion) were identified in 115 patients (115/520). Overall, 196 out of 520 patients had tier 1 or tier 2 gene alterations, and 115 of 196 had tier II gene alterations (**Supplementary File 1**).

In the second phase (Molecular epidemiology investigation), a total of 17,841 pan-cancer patients across 16 common tumor types were analyzed to evaluate the frequency of tier I and tier II gene alterations based on the genetic profiling of each patient. The druggable gene alterations included *ALK, BRAF, BRCA1/2, CDK* pathway *(CDK4/6, CDKN2A/2B), EGFR, ERBB2, FGFR1/2/3, MET, PIK3CA, RET*, and *ROS1*.

### Patient characteristics in the treatment phase

A total of 27 patients with tier II gene alterations received targeted therapy on the basis of molecular profiles. Clinical characteristics of patients receiving targeted therapy are reported in **Table 1**. The patients had received a median of 3 prior lines (range 0 - 6) of treatment for advanced disease (0−6). All patients were treated with single-agent targeted therapy; 6 patients had colorectal cancer, 8 patients had gastric cancer, and the remaining patients had cholangiocarcinoma (n = 1), scrotal Paget’s disease (n = 1), peritoneal carcinoma (n = 1), cervical cancer (n = 1), sarcoma (n = 1), nasopharyngeal carcinoma (n = 1), prostate cancer (n = 1), lung cancer (n = 1), esophageal carcinoma (n = 1), primary cancer of unknown origin (n = 1), and pancreatic ductal adenocarcinoma (n=1). The most frequent targeted alterations were *ALK* fusions, *ERBB2* mutations, and amplification, *PIK3CA* mutations, cell cycling pathway mutations (i.e., *CDK4* amplification, *CDK6* amplification, *CDKN2A* deletions), *MET* amplification, *BRCA1* mutations, and *EGFR* amplification. The most frequently used targeted agents were pyrotinib for *HER2* alterations, crizotinib for *ALK* fusions and *MET* amplification, everolimus for *PIK3CA* mutations, nimotuzumab for *EGFR* amplification, olaparib for *BRCA1* mutations and Osimertinib for *EGFR* mutation.

**Table 1:** Clinical characteristics of 27 patients receiving molecular-guided targeted therapy.

Parameters	(N=27)
**Age at inclusion**	
Median	57
Range	24-89
**Gender**	
Male	14
Female	13
**ECOG performance status**	
1	1
2	21
3	5
**Number of prior therapies**	
Median	3
Range	0-6
**Tumor Type**	
Colorectal Cancer	8
Gastric Cancer	6
Cholangiocarcinoma	1
Scrotal Paget’s Disease	1
Peritoneal Cancer	1
Cervical Cancer	1
Sarcoma	2
Nasopharyngeal carcinoma	1
Prostate cancer	1
Non-small Lung Cancer	2
Esophageal Cancer	1
Cancer of unknown primary	1
Pancreatic ductal adenocarcinoma	1
**Molecular Alteration**	
ERBB2 amplification/mutation	8
BRCA1 mutation	1
ALK fusion	4
EGFR amplification	2
PIK3CA mutation	1
CDKN2A/B mutation	4
CDK4/6 amplification	1
MET amplification	3
EGFR mutation	1

### Efficacy of molecular-targeted therapy

**Figure 2:**
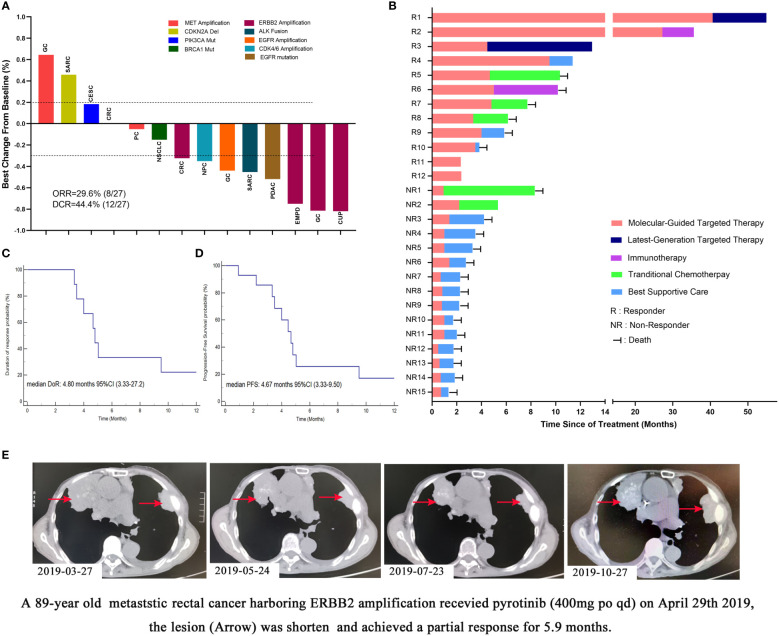
Efficacy of molecular-guided targeted therapy among Chinese intractable cancer patients. **(A)** Waterfall plots of treatment response in 14 patients (another 13 patients had been discontinued from treatment for cancer-related death or poor performance before the first response evaluation). **(B)** Follow-up of all 27 patients (R, responder, including partial respond and stable disease; NR, non-responder). **(C)** Duration of response of molecular-guided targeted therapy. **(D)** Progression-free survival of molecular-guided targeted therapy. **(E)** An 89-year-old patient with metastatic rectal cancer harboring ERBB2 amplification received pyrotinib (400 mg po qd) and achieved partial response for 5.9 months. GC, gastric cancer; SARC, sarcoma; CESC, cervical squamous cell carcinoma; PC, peritoneal cancer; NSCLC, non-small cell lung cancer; CRC, colorectal cancer; NPC, nasopharyngeal carcinoma; EMPD, extramammary Paget’s disease; CUP, cancer of unknown primary.

Among the 27 patients receiving targeted therapy on the basis of molecular profiles, 13 patients discontinued treatment due to cancer-related death or poor performance status before the first response evaluation; the patients were identified as non-responders. No patient was dead due to the toxicity of targeted therapy. The median duration of follow-up was 8.3 months (range 2.4–55.0 months). Among the additional 14 patients who received response evaluations, 8 patients showed a partial response, and 4 patients showed stable disease. Overall, ORR was 29.6% (8/27) and DCR was 44.4% (12/27**) (** **Figure 2A** **)**. **Figure 2B** showed the follow-up of all 27 patients, several responders have long survival, while median DoR was 4.80 months (95% CI, 3.33−27.2) (**Figure 2C**), and median PFS was 4.67 months (95% CI, 3.33−9.50) (**Figure 2D**). PFS1 was available in 22 patients, and 5 patients (22.7%) showed a PFS2/PFS1 ratio of >1.3 without progressive disease or death under matched targeted therapy during follow-up. **Figure 2E** showed an 89-year-old patient with metastatic rectal cancer harboring ERBB2 amplification received pyrotinib (400 mg po qd) and achieved partial response for 5.9 months.

### Frequency of off-label gene alterations in pan-cancer patients

**Figure 3:**
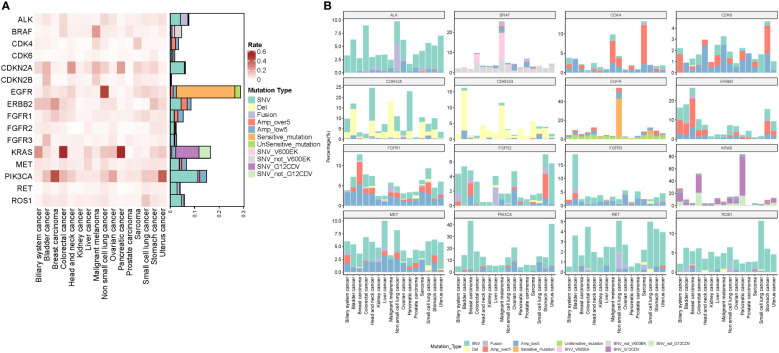
**(A)** Recurrent somatic alterations across common tumor types. According to the targeted drug, gene amplifications were divided into high-level (copy number of >5) and low-level (copy number of <5) amplifications. *EGFR* mutations were divided into sensitive and insensitive mutations. *BRAF* mutations were divided into *BRAF V600E/K* and non-*BRAF V600E/K* mutations. *KRAS* mutations were divided into *KRAS G12C/D/V* and non-*G12C/D/E* mutations. **(B)** The frequency of the druggable gene alterations in 17,841 pan-cancer patients across 16 common tumor types including *ALK*, *BRAF, BRCA1/2, CDK* pathway *(CDK4/6, CDKN2A/2B), EGFR, ERBB2, FGFR1/2/3, MET, PIK3CA, RET*, and *ROS1*.

A total of 17,841 pan-cancer patients were included to assess the frequency of off-lable gene alterations (**Table 2**). The recurrent somatic alterations across 16 common tumor types are presented in **Figure 3A**. Among these potential druggable alterations, *EGFR, KRAS*, and *PIK3CA* gene alterations were the most common, while *CDK* pathway *(CDK4/6, CDKN2A/2B), FGFR1/2/3, BRAF, RET* was uncommon across pan-cancer. The frequency of the 16 druggable gene alterations across 16 common tumor types were shown in **Figure 3B**. EGFR mutation, ALK fusion, ROS1 fusion, RET fusion was the most common in NSCLC patients. BRAF alteration were most observed in melanoma and colorectal cancer. *KRAS* mutations were observed in colorectal or pancreatic cancer patients, and *PIK3CA* mutations mainly occurred in breast cancer and uterine cancer. (**Supplementary Files 2**, **3**).

**Table 2:** Clinical characteristics of 17,841 advanced pan-cancer patients.

Patient characteristics	N	%
Total	17,841	
Gender		
Female	8,033	45.0
Male	9,279	52.0
Unknown	529	3.0
Age		
Median	60	
Range	1-94	
Tumor type		
Non-small Cell Lung Cancer	10,395	58.3
Colorectal Cancer	1,589	8.9
Breast Carcinoma	1,342	7.5
Stomach Cancer	1,106	6.2
Ovarian Cancer	592	3.3
Sarcoma	409	2.3
Pancreatic Cancer	361	2.0
Liver Cancer	320	1.8
Small Cell Lung Cancer	301	1.7
Uterus cancer	482	2.7
Kidney Cancer	210	1.2
Head and neck Cancer	203	1.1
Biliary System Cancer	183	1.0
Melanoma	122	0.7
Prostate Cancer	115	0.6
Bladder Cancer	111	0.6

**Figure 4:**
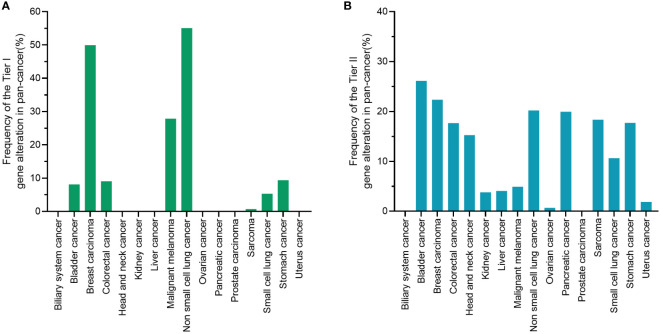
Tier I or tier II alterations in 17,841 pan-cancer patients. **(A)** Tier I variants are of strongest clinical significance, and include level A evidence (FDA-approved therapy included in professional guidelines) and level B evidence (well-powered studies with consensus from experts in the field). **(B)** Tier II alterations reflect the possibility of off-label use of a targeted drug in pan-cancer patients. A tier II variant was defined as a variant of potential clinical significance, and included level C evidence (FDA-approved therapies for different tumor types or investigational therapy, multiple small published studies with some consensus) and level D evidence (preclinical trials or a few cases reports without consensus). FDA, food and drug administration.

**Figure 4** displays the frequency of tier I and tier II alterations in 17,841 pan-cancer patients. The frequency of tier I gene alterations in pan-cancer patients was 37.6% (**Figure 4A**). NSCLC patients harbored the most tier I gene alterations (55.1%), followed by breast cancer (49.9%), malignant melanoma (27.9%), and colorectal cancer (9.1%). After the tier I gene alterations were excluded, only tier II alterations with the possibility of off-label use of a targeted drug in pan-cancer patients remained. **Figure 4B** shows the tier II gene alterations among 17,841 pan-cancer patients. Overall, the frequency of tier II alterations in pan-cancer patients was 17.7%, suggesting that 17.7% of pan-cancer patients had the potential to receive a molecular-guided targeted therapy. Bladder cancer had the most tier II gene alterations (26.1%), followed by breast cancer (22.4%), NSCLC (20.2%), pancreatic cancer (19.9%), and sarcoma (18.3%). Biliary system and prostate cancer showed the lowest number of tier II alterations.

## Discussion

The Long March Pathway was a basket trial designed to evaluate the efficacy of targeted therapy on the basis of molecular profiles for intractable cancer harboring tier II gene alterations. A total of 27 of 115 patients with tier II alterations received targeted therapy with an ORR of 29.6%, DOR of 44.4%, and PFS of 4.67 months. The molecular profiles of 17,841 pan-cancer patients showed that the frequency of tier II alterations across cancer types was 17.7%, indicating that 17.7% of patients could benefit from off-label molecular-guided targeted therapy.

Patients with intractable cancer do not respond well to standard chemotherapeutic regimens after multiple-line therapy, or cannot tolerate standard of care due to poor health conditions (12). In the era of genomics driving decision making in oncology, basket trials may provide guidance for the use of highly effective, and low-toxicity targeted therapy for these patients. However, there are two factors that warrant consideration prior to the application of targeted therapy to intractable cancer. First, it must be determined whether intractable cancer patients harbor specific druggable molecular alterations, especially for tier II gene alterations that do not have a currently available FDA indication. Second, it must be determined whether a molecular-guided targeted therapy will still be efficacious in intractable cancers with specific gene alterations.

To address the first point, the investigators have previously performed several studies. The ProfiLER study showed that 52% patients had at least 1 actionable mutation across 16 refractory cancer types (25). Similarly, SHIVA study found that 40% of refractory cancer patients had at least 1 druggable molecular alteration (26). The MOSCATO-01 study demonstrated that 411 out of 843 (49%) patients with advanced hard-to-treat cancers, and 42 out of 69 (60.9%) pediatric patients had actionable molecular alterations (27, 28). With respect to rare cancers, Kato et al. (29) reported that 37 patients (92.5%) had at least 1 potentially actionable target among 40 patients who underwent genomic and protein analyses (30). Meanwhile, the genetic profiling of 5,954 refractory malignancies from the National Cancer Institute Molecular Analysis for Therapy Choice (NCI-MATCH) trial showed that 37.6% cases had an actionable alteration. Although there are some discrepancies among the reported frequencies, all these data demonstrated the existence of druggable molecular alterations among intractable cancers. As previous studies reported, the genetic landscape, and response to targeted therapy, vary across ethnicities (31). The molecular profiles of Chinese cancer patients remain unclear. Consistent with previous studies, this study demonstrated that 22.1% (115 of 520) of Chinese intractable cancer have at least 1 tier II alteration. Collectively, these data indicate the feasibility of molecular-guided targeted therapy and a high demand for this innovative and efficient treatment strategy for intractable cancers.

To further explore the possibility of molecular-guided targeted therapy across cancer types, the mutational landscape of metastatic cancer was analyzed using an NGS panel (256 or 520 gene panel) in 17,841 Chinese pan-cancer patients to detect the frequencies of druggable targets. The frequency of tier II gene alterations across pan-cancer outside of current FDA indications was 17.7%, suggesting that clinicians or physicians can consider various molecular targeted therapies across different tumor types with shared genetic features for the treatment of patients, especially for intractable cancer. In 2017, the mutational landscape of metastatic advanced cancer in 10,366 patients from the Memorial Sloan Kettering Cancer Center (MSKCC) was published. The study demonstrated that 27% of patients had at least 1 actionable alteration apart from targets with levels 1 and 2A evidence according to whether mutations were FDA-recognized biomarkers. On comparing the distribution of actionable alterations between the MSCKCC and Long March Pathway cohorts, it was evident that while gynecological cancer and biliary cancer in the Chinese population harbored fewer alterations, lung cancer patients had more alterations than the MSKCC cohort. Some types of cancer, including breast cancer and bladder cancer, showed higher frequency of druggable targets in both cohorts Similarly, some gene alterations, including *KRAS and PIK3CA* mutations, were shared among different ethnicities, while some alterations, including *EGFR* mutation (enriched in Chinese NSCLC patients) were variably represented. This variation in the distribution of gene alterations thus warrants further investigations in other ethnic populations.

Previous studies have shown that targeted therapy may elicit different responses among patients from different histology. For example, melanoma patients harboring *BRAF V600E* were found to respond well to vemurafenib, while those with colorectal cancer did not (32, 33). Similarly, recent trials have shown that colorectal cancer and NSCLC seem to respond differently to AMG510 although these cancers share *KRAS G12C (* 34). Thus, whether targeted therapy, regardless of histology, are still efficacious in intractable cancers according to basket trials is yet to be fully elucidated, and controversies regarding the role of targeted therapy decisions based on genomics in intractable cancers warrants further investigation. Some studies have provided validation for this strategy in clinical practice, and that molecular-targeted cancer therapy based on basket trials may yield survival benefits in patients with intractable cancers (13, 14, 23, 25, 27, 35). In addition, molecular-targeted cancer therapy has also resulted in improvements in prognosis among patients with rare cancers with no recommended regimen, younger cancer patients, and patients with refractory cancers of unknown origin (30, 36, 37). However, findings from some studies evaluating the efficacy of genomics-guided targeted therapy in intractable cancer are conflicting. The NCI-MATCH (EAY131) trial was the largest multiple basket trial study conducted to date that aimed to determine whether specific targeted therapies may benefit a greater number patients and lead to more personalized therapies in advanced cancer patients without standard treatment options (38). The EAY131-H study demonstrated an ORR of 38% after treatment of 29 pretreated, pan-cancer patients with mixed histology with dabrafenib and trametinib (39). However, the EAY131-Q study evaluated the efficacy of Ado-trastuzumab emtansine (T-DM1) in heavily pre-treated pan-cancer patients harboring HER2 amplification. The study noted an ORR rate of just 5.6% (40). The EAY131-W study found that the use of AZD4547, an oral FGFR1-3 inhibitor in patients with refractory cancer harboring aberrations in the FGFR pathway did not meet the primary endpoint (response rate of 16%) (41). Furthermore, the SHIVA study also demonstrated that the use of molecular-targeted therapy outside the approved indications did not significantly improve PFS in heavily treated cancer patients (42). There are several factors that may explain why this controversy regarding whether the use of genomics-guided therapy can improve outcomes in intractable cancer, including differences in targeted agents, oncogenic driving capacity, tumor heterogeneity, tumor genetic background, etc. (43–45). Although a common set of driver mutations exist within each cancer type, the combination of drivers and their distribution within the founding clone and subclones vary across patients, which could affect the efficacy of targeted therapy. This suggests that knowledge of the tumor’s clonal architecture is crucial for optimizing treatment. Meanwhile, the genetic backgrounds across different ethnicities may affect tumor-related molecular alterations. Molecular alteration frequency and response to targeted therapy also vary among across different ethnicities. Thus, the Long March Pathway trial conducted in the Chinese population is important and supports further genomics-guided targeted therapy in intractable cancer.

Our results have validated the use of targeted therapy on the basis of molecular profiles in clinical practice and have shown that the discussed methods can help guide treatment choices for intractable cancer. However, several limitations require clarification for accurate interpretation of the results. First, the ratio of patients who received off-label targeted therapy was low, and 23.5% (27 of 115) of patients received targeted therapy based on an individual molecular profile. The low ratio may be attributed to the high cost of targeted therapy and off-label treatment, which medical insurance does not cover. Second, a few of patients (48.1%, 13/27) failed to undergo response evaluation. Most patients discontinued the targeted therapy due to poor performance or cancer-related death during treatment. These patients were defined as non-responders to off-label targeted therapy. This might have affected the accuracy of efficacy evaluation. Third, the small sample size limited the efficacy evaluation of different druggable targets. For example, only 8 targeted therapies were assessed under multiple basket trials. The efficacy of molecular-targeted therapy against another 5 druggable targets (*BRAF* mutations, *FGFR* alterations, *RET* fusions, and *ROS1* fusions) could not be evaluated, while the association of targeted agents type and the concomitant alterations with a response to targeted therapy were also not further assessed (46). In addition, the treatment regimen was not the current optimal standard care (e.g., crizotinib for *ALK* fusion, single-agent vemurifinib for *BRAF V600E*). Thus, the further exploration of the application of basket trials in a larger patient cohort is recommended.

In conclusion, the Long March Pathway trial demonstrated a significant clinical benefit for intractable cancers using targeted therapy based on molecular profiles in the Chinese population. The frequency of tier II gene alterations across a variety of cancers supports the feasibility of molecular-guided targeted therapy under basket trials and may provide novel insights into the optimal clinical treatment of intractable cancers.

## Data availability statement

The original contributions presented in the study are included in the article/**Supplementary Material**. Further inquiries can be directed to the corresponding author.

## Ethics statement

The studies involving human participants were reviewed and approved by The Ethics Committee of Changzheng Hospital. The patients/participants provided their written informed consent to participate in this study.

## Author contributions

X-DJ and Y-SZ designed the study. X-DJ, B-DQ, ZW, KL, YW, YL, W-XQ, M-MW, L-YY, HL, Y-YX, X-MQ, MW, MJ, L-CX, YZh, HY, X-QC, YZe, JS, W-YZ, Q-QD, WJ, S-FL, J-JZ, HS, S-HY, Z-JG, TL, P-JZ, X-WD, and W-FL recruited patients for this study and collected data. X-DJ, B-DQ, ZW, KL, YW, YL, W-XQ, M-MW, L-YY, and Y-SZ did the analyses. X-DJ, B-DQ, SB, AK, KM, RC, MA, CA, FP, and Y-SZ interpreted the data and wrote the manuscript. All authors have agreed to be accountable for all aspects of the work in ensuring that questions related to the accuracy or integrity of any part of the work are appropriately investigated and resolved. All authors contributed to the article and approved the submitted version.

## Supplementary material

Supplementary File 1 The frequency of tier 1 and tier 2 gene alterations in 520 intractable cancer patients.

Supplementary File 2 The frequency of the druggable gene alterations, including ALK, BRAF, BRCA1/2, CDK pathway (CDK4/6, CDKN2A/2B), EGFR, ERBB2, FGFR1/2/3, MET, PIK3CA, RET, and ROS, in different types of cancer.

Supplementary File 3 Raw data on the frequency of the druggable gene alterations in 17,841 pan-cancer patients across 16 common tumor types
