# Supplementary figures and images for: Targeted therapy for intractable cancer on the basis of molecular profiles: An open-label, phase II basket trial (Long March Pathway)
Source: Front Oncol. 2023 Feb 23;13:860711. doi: 10.3389/fonc.2023.860711 (PMC9995917; doi:10.3389/fonc.2023.860711)

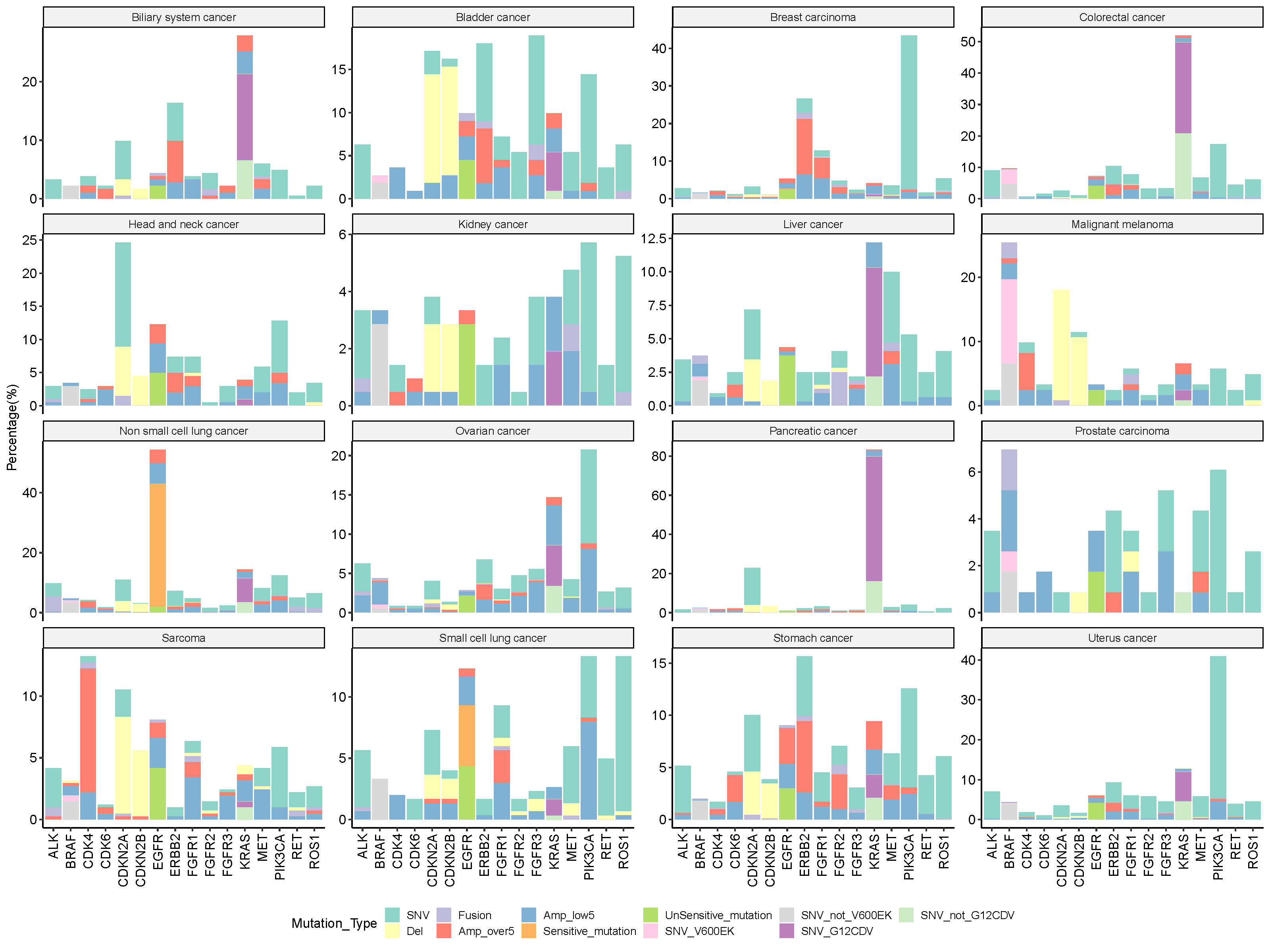

Supplement: Supplementary File 1 — The frequency of tier 1 and tier 2 gene alterations in 520 intractable cancer patients. [file Image_1.tiff]
